# SRF SUMOylation modulates smooth muscle phenotypic switch and vascular remodeling

**DOI:** 10.21203/rs.3.rs-2922216/v1

**Published:** 2023-05-22

**Authors:** Yue Xu, Haifeng Zhang, Jordan S. Pober, Wang Min, Jenny Huanjiao Zhou

**Affiliations:** 1 Interdepartmental Program in Vascular Biology and Therapeutics, Department of Pathology, Yale University School of Medicine, New Haven, CT.; 2 Yale Center for Genome Analysis, Cancer Department of Genetics, Yale University School of Medicine, New Haven, CT.

**Keywords:** Vascular remodeling, neointima, smooth muscle cell, SUMOylation, SRF, ELK1

## Abstract

Serum response factor (SRF) controls gene transcription in vascular smooth muscle cells (VSMCs) and regulates VSMC phenotypic switch from a contractile to a synthetic state, which plays a key role in the pathogenesis of cardiovascular diseases (CVD). SRF activity is regulated by its associated cofactors. However, it is not known how post-translational SUMOylation regulates the SRF activity in CVD. Here, we show that *Senp1* deficiency in VSMCs increased SUMOylated SRF and the SRF-ELK complex, leading to augmented vascular remodeling and neointimal formation in mice. Mechanistically, SENP1 deficiency in VSMCs increased SRF SUMOylation at lysine 143, which reduced its lysosomal localization concomitant with increased nuclear accumulation. SUMOylation of SRF switched its binding with the contractile phenotype-responsive cofactor myocardin to binding with the synthetic phenotype-responsive cofactor phosphorylated ELK1. Both SUMOylated SRF and phosphor-ELK1 were increased in VSMCs from coronary arteries of CVD patients. Importantly, preventing the shift from SRF-myocardin to SRF-ELK complex by AZD6244 inhibited the excessive proliferative, migratory, and synthetic phenotypes, attenuating neointimal formation in *Senp*1-deficient mice. Therefore, targeting the SRF complex may have a therapeutic potential for the treatment of CVD.

## INTRODUCTION

The regulation of vascular smooth muscle cell (VSMC) fate is involved in vascular development and pathological vascular remodeling in human cardiovascular diseases (CVD)^1,2^. VSMCs reside in the media of the vascular wall in a relatively differentiated state characterized by abundant expression of contractile phenotype-associated genes, including CNN1, α-SMA, SM-22α, and MYH11^2^. In response to vascular injury or platelet-derived growth factor (PDGF)-BB stimulation, VSMCs undergo phenotypic switching from contractile phenotype to a dedifferentiated synthetic state, accompanied by decreased contractile phenotypeassociated gene expression and increased expression of genes associated with proliferation, migration, and synthetic markers (MYH10 and OPN) that constitute part of the pathological processes of vascular remodeling^1–3^. Thus, understanding the molecular mechanisms governing VSMC phenotypic switch may provide novel avenues for interventions in vascular remodeling after injury.

VSMC fate is controlled by several transcriptional regulators^4^. Serum response factor (SRF) is a master switch that regulates VSMC contractility^4–6^. Under normal condition, SRF binds to CArG box [(CC(A/T)_6_GG) elements, together with its cofactors, myocardin (MYCD) and myocardin-related transcriptional factor-A (MRTFA), to maintain VSMC contractile phenotype^4–7^. However, it is now appreciated that the two SRF cofactors have opposing roles in vivo. Specifically, whereas MYCD is the gate-keeper for a normal contractile state, upregulated MRTFA in pathogenesis promotes vascular pathological vascular remodelling^8,9^. Moreover, vascular injury or PDGF-BB stimulation induces MEK-extracellular signal-related kinases (ERK)-dependent phosphorylation of Ets-like transcription factor-1 (ELK1), that displaces SRF from the myocardin complex to form the SRF-ELK1 complex which binds to an adjacent ternary complex element (TCE), resulting in VSMC contractile phenotypic switch to a synthetic state^4–7,10^. In contrast to the relatively well-studied transcriptional control mechanisms, regulation of VSMC fate at the post-transcriptional level, such as SUMOylation, has not been well investigated.

SUMOylation is a reversible and dynamic protein post-translational modification by the small ubiquitinlike modifier (SUMO) proteins, that modulates protein activity, stability, subcellular localization and protein-protein interactions^11,12^. SUMOylation is involved in a wide variety of cellular processes, such as transcription, DNA repair, trafficking, and signal transduction^13^. The SUMO family in mammals has three analogs, SUMO1, SUMO2 and SUMO3, that are covalently attached to specific lysine(s) of target proteins. SUMOylation involves a multistep enzymatic cascade, sequentially catalyzed by activating (E1), conjugating (E2), and ligating (E3) enzymes, that facilitates SUMO attachment to its substrates^13,14^. Being reversible, SUMOylation is readily deconjugated by a seven-member family of SUMO-specific proteases (SENPs): SENP 1–3 and 5–8^12,15^. Among these, SENP1 is ubiquitously expressed, localized in the nucleus and other discrete cellular compartments, and largely responsible for deconjugating SUMO1 and SUMO2/3 modifications^15^.

Previous studies^16–19^, including our work^20–25^ demonstrated that SENP1 deconjugates several SUMOylated proteins and is, thus, involved in many metabolic stress-, inflammatory response-, vascular disease- and tumor-related cellular processes. Global *Senp1* deletion in mice causes perinatal lethality due to defective erythropoiesis^20,26^. We previously reported delayed vascular formation in *Senp1* global knockout mice^20^. Moreover, SENP1-mediated SUMOylation in vascular endothelial cells (ECs) has been extensively investigated in EC-specific *Senp*1-deficient mice; under physiological and pathological conditions, SENP1 targets various substrates, including membrane receptors (Notch and VEGFR2) and nuclear transcriptional factors (GATA2 and NF-kB), to regulate angiogenesis and arteriosclerosis^22,23^. Moreover, we recently reported that *Senp*1 deficiency in SMCs induces ERα SUMOylation, which augments ERα transcriptional activity and stromal CD34^+^ KLF4^+^ stem cell proliferation, thereby promoting endometrial regeneration and repair^24^. However, the role of SENP1-mediated SUMOylation in VSMCs is unclear. The present study aimed to identify a direct association between SENP1-mediated SUMOylation and VSMC fates in vascular remodeling and human CVD.

## RESULTS

### Senp1 deficiency in VSMCs accelerates injury-induced neointimal formation with enhanced proliferation and migration

Partial loss of SENP1 protein and mRNA expression in carotid arteries of *Senp*1^ECKO^ mice, and near complete loss in *Senp*1^SMCKO^ mice, were observed due to abundant VSMCs in the aorta (Fig.S1A-B). Immunohistochemistry analysis further revealed specific ablation of the SENP1 protein in VSMCs of *Senp*1^SMCKO^ mice and ECs of *Senp*1^ecKO^ mice (Fig.S1C).

**Fig. 1.**
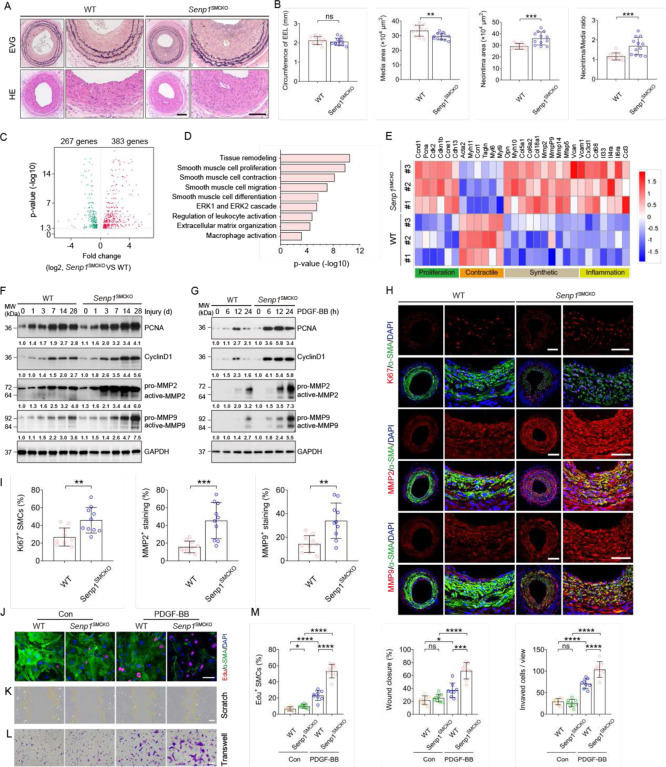
*Senp*1 deficiency in VSMCs accelerates injury-induced neointimal formation with enhanced VSMCs proliferation and migration. *Senp*1^lox/lox^ (WT) and *Senp*1^SMCKO^ mice at age of 10–12 weeks were subjected to wire injury on left carotid arteries, and tissues were harvested for analyses on day 3–28 postinjury as indicated. Non-injured mice were used as controls (time 0). **A**. Representative photomicrographs of EVG staining and HE staining of carotid arteries harvested on day 28 post-injury from WT and *Senp1*^SMCKO^ mice. **B**. Circumference of EEL, neointimal area, media area, and neointima/media ratio in carotid arteries were measured (n=12 per group). **C–E**. Total RNAs were isolated from carotid arteries on day 14 post-injury and were subjected to RNA-sequencing analyses (n=10 per group). (**C**) Volcano plot of the differentially expressed mRNAs between WT and *Senp*1^SMCKO^ mice. (**D**) GO analysis showing the biological functions related with the differentially expressed genes in carotid arteries of WT and *Senp1*^SMCKO^ mice. (**E**) Heat map showing the gene expression of proliferation, contractile phenotype, synthetic phenotype, and inflammation in carotid arteries of WT and *Senp*1^SMCKO^ mice. **F–G**. Western blots for proteins of VSMC proliferation and migration markers in carotid arteries of WT and *Senp*1^SMCKO^ mice harvested on day 0–28 post-injury (F) or VSMCs isolated from non-injured WT and *Senp*1^SMCKO^ mice treated with PDGF-BB (10 ng/ml) for indicated times(G). Relative protein levels are presented by taking non-injured WT as 1.0 (n=3 per group). **H–I**. Carotid arteries on day 28 post-injury were subjected to immunofluorescence co-staining of α-SMA (green) with Ki67, MMP2 or MMP9 (red) with DAPI counterstaining for nuclei (blue). % of Ki67^+^α-SMA^+^ VSMCs, MFI of MMP2 and MMP9 within the neointimal area was quantified (n=10 per group). **J–M**. VSMCs isolated from WT and *Senp*1^SMCKO^ mice were subjected to PDGF-BB-induced proliferation and migration assays. (**J**) Edu incorporation: Co-staining of α-SMA by immunostaining and EdU by a Click-iT assay. (**K**) Scratch assay was performed in the absence or presence of PDGF-BB for 24 h before fixation and imaging. (**L**) Transwell migration assay was performed in the absence or presence of PDGF-BB for 24 h before fixation and staining with crystal violet. (**M**) % Edu^+^α-SMA^+^ in (**J**), % wound closure in (**K**) and number of invaded VMSCs in (**L**) were quantified (n = 8 per group). Data are mean ± SEM. * P < 0.05; ** P < 0.01; *** P < 0.001; **** P < 0.0001; ns, no significance, using unpaired, two-tailed Student’s t-test (B, I), one-way ANOVA followed by Tukey’s multiple comparisons test (M). Scale bars, 50 μm (A, H); 10 μm (J, K, L). MFI: mean fluorescence intensity.

At baseline conditions at 10–12 weeks of age, the external elastic lamina (EEL) circumference, luminal area, and media area of carotid artery did not differ between WT and *Senp*1^SMCKO^ mice as determined by EVG and HE staining (Fig.S2A-B). To determine the role of SENP1 in vascular remodeling, we performed wire injury in the carotid artery, a widely used method to study mechanisms of vascular remodeling^27^ in WT and *Senp*1^SMCKO^ mice. *Senp*1 deficiency in VSMCs significantly aggravated injury-induced neointimal formation 28 days after wire injury, reflected by enlarged intima areas, decreased media areas, and increased intima/media ratios. The EEL circumference did not differ between WT and *Senp*1^SMCKO^ mice (Fig.1A with quantifications in B). These results indicate that *Senp*1 deficiency in VSMCs accelerates injury-induced neointimal formation. This is in sharp contrast to our previous observation in *Senp1*^ECKO^ mice where EC-specific deletion attenuated vascular remodeling in several models^22,23,25^, suggesting that SENP1 has distinct regulatory functions in VSMCs and aortic ECs.

To elucidate SENP1 regulation of vascular remodeling, we first performed whole-transcriptome analysis by a bulk RNA-seq of carotid arteries of *Senp*1^SMCKO^ and WT mice at basal and at 14 days after wire injury. RNA-seq analysis revealed that VSMC-specific *Senp1* deletion resulted in significant downregulation of 267 genes and upregulation of 383 genes in the carotid artery (Fig.1C). Gene ontology (GO) analysis revealed that *Senp1* deletion significantly enriched physiological processes related to tissue remodeling, VSMC proliferation, contraction, migration, differentiation, ERK1/2 cascade, regulation of leukocyte activation, extracellular matrix (ECM) organization, and macrophage activation (Fig.1D). Further analysis of these mRNAs revealed that VSMC-specific *Senp1* deletion decreased the markers of contractile phenotype (*Acta*2, *Myh*11, *Ccn*1, *Tagln*, *Myl*6, and *Myl*9) and increased the markers of proliferation (*Ccnd*1, *Pcna*, *Cdk*2, *Cdkn*1b, *Ccne*1, *Cdh*13), synthetic phenotype (*Opn*, *Myh*10, *Col5*a1, *Col6*a2, *Col*18a1, *Mmp*2, *Mmp*9, *Mmp*14, *Mfap*5, and *Vcan*), and inflammation (*Vcam*1, *Cx3cl*1, *Cd*68, *Il*33, *Il*4ra, *Il*6ra, and *Ccl*3) (Fig.1E). Despite the morphometry of carotid artery at baseline condition was not altered by VSMC-specific *Senp1* deletion, significant gene expression changes were detected in *Senp*1^SMCKO^ compared to WT aortas, with reduced contractile marker genes and increased synthetic marker and inflammatory genes (Fig.S2C). Collectively, these results indicate a pivotal role of SENP1 in regulating VSMC proliferation, migration, and phenotypic switch.

As VSMC proliferation and migration are key processes in neointimal formation in response to arterial injury or mitogenic factors, we investigated the potential effect of SENP1 on both processes. Western blot analysis revealed upregulated expression of the proliferation inducing proteins cyclin D1 and proliferating cell nuclear antigen (PCNA) in response to injury in a time-dependent manner; Significantly higher cyclin D1 and PCNA levels were detected in *Senp1*^SMCKO^ mice than that in WT mice (Fig.1F). Similar to the in vivo observations, cultured aortic VSMCs from *Senp1*^SMCKO^ mice exhibited higher cyclin D1 and PCNA expression after PDGF-BB treatment compared with VSMCs from WT mice (Fig.1G). Furthermore, immunohistochemical assays for Ki67, a proliferation marker, showed that *Senp*1 deficiency in VSMCs dramatically enhanced their proliferation, as determined by markedly increased Ki67- and α-SMA-positive VSMCs compared with WT mice, 28 days after wire injury (Fig.1H). Matrix metalloproteinase (MMP)-facilitated VSMCs migration from the media to the intima is critical for postinjury neointimal formation^28^. Western blot demonstrated that wire injury or PDGF-BB treatment induced MMP2 and MMP9 protein activity, with significantly higher levels in *Senp1*^SMCKO^ mice than WT mice (Fig.1F–G). Consistently, immunohistochemical assays showed dramatically higher MMP2 and MMP9 protein expression levels in carotid arteries of *Senp1*^SMCKO^ mice compared with WT mice 28 days after wire injury (Fig.1H–I). Moreover, multiple ECM-related genes required for VSMC migration (*Col*15a1, *Col*5a2, *Col*6a2, and *Col1*a1), tissue inhibitor of matrix metalloproteinase 1 (*Timp*1), and *Mmp*14 were upregulated after wire injury as determined by qRT-PCR. *Senp*1 deficiency in VSMCs significantly aggravated injury-induced expression of Col15a1, Col5a2, Col6a2, and Timp1 (Fig.S3).

We further evaluated VSMC proliferation and migration using in vitro models. To this end, PDGF-BBstimulated aortic VSMCs from WT an *Senp1*^SMCKO^ mice were subjected to Edu incorporation assays. *Senp*1deficient VSMCs proliferated more than WT VSMCs (Fig.1J with quantifications in 1M for Edu^+^ SMCs). Transwell and scratch assays revealed that *Senp*1 deletion increased PDGF-BB-induced VSMC migration (Fig.1K, L with quantifications in 1M for wound closure and invasion cells).

We further investigated whether *Senp*1 deficiency-mediated induction of neointima growth in VSMCs is related to decreased apoptotic activity. Western blot analyses found no difference in the expression levels of apoptosis-related genes (caspase-9, Bcl-2, and Bax) in *Senp*1^SMCKO^ mice compared with WT mice (Fig.S4A). Immunohistochemical assay of cleaved caspase-3, another pro-apoptotic marker, showed no difference in *Senp*1^SMCKO^ mice compared with WT mice 28 days after injury (Fig.S4B-C). Taken together, these results indicate that *Senp*1 deficiency in VSMCs promotes their proliferation and migration, thereby accelerating injury-induced neointimal formation.

### Senp1 deficiency in VSMCs results in loss of contractile phenotype in injury-induced neointima

**Fig. 2.**
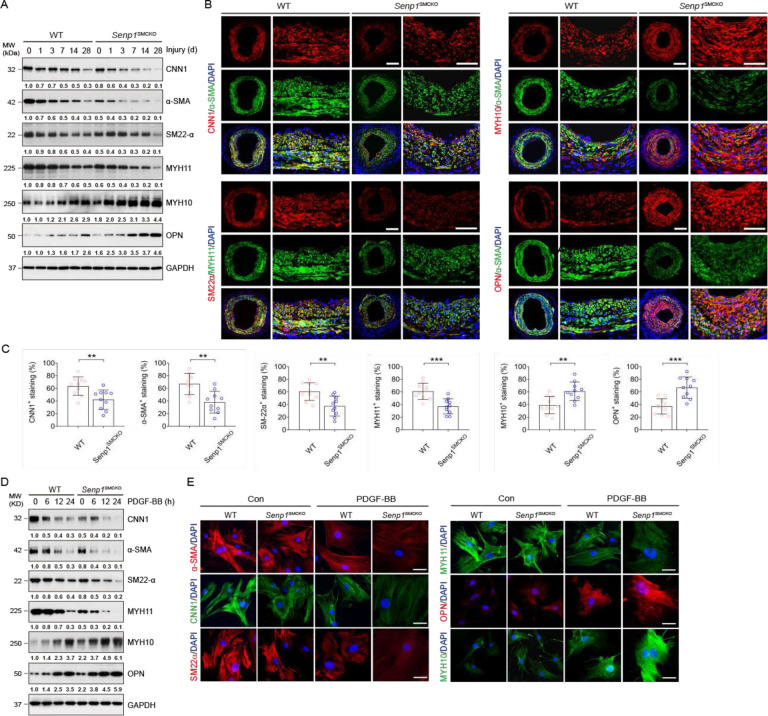
SENP1 deficiency in VSMCs results in a loss of contractile phenotype in injury-induced neointima. WT and *Senp*1^SMCKO^ mice at age of 10–12 weeks were subjected to wire injury on left carotid arteries, and tissues were harvested for analyses on day 3–28 post-injury as indicated. Non-injured mice were used as controls (time 0). **A**. Western blots for proteins of VSMC contractile and synthetic markers on day 028 post-injury. Relative protein levels are presented by taking non-injured WT as 1.0 (n=3 per group). **B–C**. Carotid arteries on day 28 post-injury were subjected to immunofluorescence co-staining of various contractile and synthetic markers as indicated with DAPI counterstaining for nuclei (blue) (B). MFI of each marker within the neointimal area was quantified (n=10 per group) (C). **D–E**. VSMCs isolated from non-injured WT and *Senp*1^SMCKO^ mice treated with PDGF-BB (10 ng/ml) for indicated times. (D) VSMC contractile and synthetic markers were detected by Western blot (0, 6, 12 and 24 h time points) and (E) immunofluorescence staining (0 and 24 h time point). Relative protein levels in (D) are presented by taking non-injured WT as 1.0 (n=3 per group). Data are mean ± SEM. ** P < 0.01; *** P < 0.001, using unpaired, two-tailed Student’s t-test (C). Scale bars, 50 μm (B). 10 μm (E). MFI: mean fluorescence intensity.

VSMC phenotype switching, from a differentiated contractile state to a dedifferentiated synthetic state, plays a vital role in neointimal formation after vascular injury^2,3^. Consistent with the bulk RNA-seq data, VSMC-specific *Senp*1 deletion weakly down-regulated basal levels of VSMC contractile markers, such as CNN1, α-SMA, SM22α, and MYH11, and up-regulated those of VSMC synthetic markers, such as MYH10 and OPN, as detected by immunostaining (Fig.S5) and western blot (Fig.2A). Moreover, wire injury caused further loss of all four contractile markers, while increasing expression of two synthetic markers in a timedependent manner. *Senp*1^SMCKO^ mice exhibited a more rapid decrease and increase in the four contractile markers and two synthetic markers, respectively, compared with WT mice (Fig.2A). This phenotypic switch within the neointima on day 28 post-injury was more evident in the *Senp*1^SMCKO^ mice (Fig.2B–C).

To validate these observations in vitro, cultured aortic VSMCs were exposed to PDGF-BB, the most widely accepted approach for initiating VSMC phenotype switch in vitro. Western blots (Fig.2D) and immunohistochemical staining (Fig.2E) showed decreased expression of contractile markers (CNN1, α-SMA, SM22α, and MYH11), while markedly increased expression of synthetic markers (MYH10 and OPN) in PDGFBB-treated cells compared to that in untreated VSMCs. *Senp*1 deficiency in VSMCs caused significant repression of CNN1, α-SMA, SM22α, and MYH11, but promotion of MYH10 and OPN (Fig.2D–E) in a timedependent manner compared to the VSMCs from WT mice. These results demonstrated that Senp1 in VSMCs is essential for the maintenance of the contractile phenotype of carotid arteries both in physiological and pathological states.

### Senp1 deficiency in VSMCs augments the SRF-ELK complex formation during vascular remodeling

**Fig. 3.**
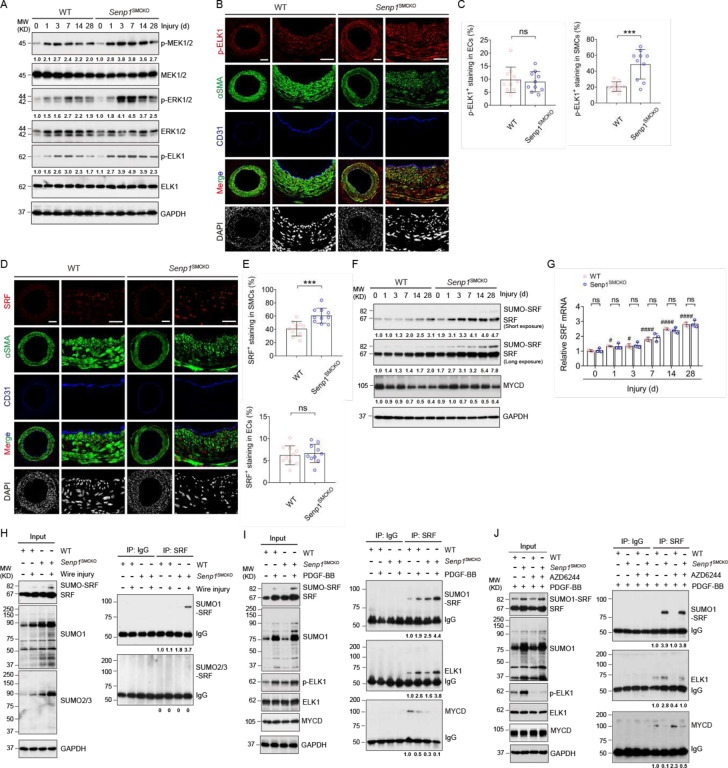
SENP1 deficiency in VSMCs augments the SRF-ELK complex during vascular remodeling. Carotid arteries from WT and *Senp*1^SMCKO^ mice on day 0–28 post-injury. **A**. Western blots for proteins in the MEK-ERK1/2-ELK1 pathway. Relative protein levels are presented by taking non-injured WT as 1.0 (n=3 per group). **B–C**. Immunofluorescence staining for p-ELK in carotid arteries from *WT* and *Senp*1^SMCKO^ mice on day 28 post-injury. Four color images are presented with p-ELK (red), α-SMA (green), CD31 (APC-conjugated; pseudo-colored by blue) and DAPI (blue; pseud-colored by white). (C) MFI of p-ELK within the neointimal areas or ECs were quantified (n =10 per group). **D–E**. Immunofluorescence staining for SRF in carotid arteries from WT and *Senp*1^SMCKO^ mice on day 28 post-injury. (D) Four color images are presented with SRF (red), α-SMA (green), CD31 (APC; pseudo-colored by blue) and DAPI (blue; pseud-colored by white). (E) MFI of SRF within the neointimal areas or ECs were quantified (n =10 per group). **F**. Western blots for SRF and myocardin. Relative protein levels are presented by taking non-injured WT as 1.0 (n=3 per group). **G**. qRTPCR for Srf mRNAs in carotid arteries from WT and *Senp*1^SMCKO^ mice. Gapdh served as a control. Relative mRNA levels are presented by taking non-injured WT as 1.0 (n=3 per group). **H**. Detection of SRF SUMOylation in day 7 post-injury carotid arteries from WT and *Senp*1^SMCKO^ mice. Artery lysates were subjected to co-immunoprecipitation assays with anti-SRF or IgG followed by western blot with SUMO1 or SUMO2/3 as indicated. Relative protein levels in the input and co-immunoprecipitates are presented by taking non-injured carotid artery from WT mice as 1.0 (n=3 per group). **I**. Detection of SRF-SUMO1, SRF-ELK and SRF-myocardin complexes. VSMCs from WT and *Senp*1^SMCKO^ mice were untreated or treated with PDGFBB (10 ng/ml for 60 min). VSMCs lysates were subjected to co-immunoprecipitation assays with anti-SRF or IgG followed by western blot with SUMO1, ELK1, and myocardin as indicated. Relative protein levels in the input and co-immunoprecipitates are presented by taking untreated VSMCs from WT mice as 1.0 (n=3 per group). **J**. Effects of AZD6244 on the SRF complexes in PDGF-treated VSMCs from *WT* and *Senp*1^SMCKO^ mice. VSMCs were treated with PGDF-BB in the absence or presence of AZD6244 (0.5 μmol/L) for 60 min. Cell lysates were subjected to co-immunoprecipitation assays with anti-SRF or IgG followed by Western blot with SUMO1, ELK or myocardin as indicated. Relative protein levels in the input and co-immunoprecipitates are presented by taking untreated VSMCs from WT mice as 1.0 (n=3 per group). Data are mean ± SEM. ns, no significance, compared with WT mice (two-tailed Student’s t-test) (C, E). # P < 0.05; #### P < 0.0001, compared with un-injured or untreated groups (One-way ANOVA with Bonferroni post hoc analysis) (G). Scale bars, 50 μm (B, D).

MEK1/2-ERK1/2-ELK1 pathway activation in VSMCs after vascular injury is responsible for ELK1mediated transcriptional switch from contractile to synthetic phenotype^5^. Phosphorylated MEK1/2, ERK1/2, and ELK1 levels were low in normal carotid arteries and cultural aortic VSMCs, but progressively increased after wire injury. *Senp1*^SMCKO^ mice exhibited a more rapid increase in phosphorylated MEK1/2, ERK1/2, and ELK1 levels compared with WT mice (Fig.3A). Immunohistochemical staining revealed a significant increase in phosphor-ELK1-positive cell number in the neointima of *Senp1*^SMCKO^ mice compared with that of WT mice 28 days after injury; most of these were co-localized with α-SMA-positive VSMCs, and little with CD31positive ECs (Fig.3B with quantifications in C). Since SRF together with its cofactor myocardin maintain VSMC contractile phenotype^4–7,10^, we also examined if their expressions were altered by SENP1 deletion. At basal conditions, SRF protein expression was significantly increased in the carotid arteries of *Senp1*^SMCKO^ mice compared with WT mice, as demonstrated by its high expression in α-SMA-positive VSMCs of carotid artery from *Senp1*^SMCKO^ mice (Fig.S6A-B). SRF-positive cell number was further increased in *Senp1*^SMCKO^ mice after vascular injury (Fig.3D–E). SRF was expressed predominantly in α-SMA-positive VSMCs, but not in CD31-positive ECs (Fig.S6A-B; Fig.3). Western blot indicated that injury indued a time-dependent increase of SRF expression in the carotid artery which was significantly augmented by SENP1 deletion (Fig.3F). The expression of myocardin was not altered in response to wire injury in both groups (Fig.3F). Despite the increases of SRF protein levels, Srf mRNA levels in the injured carotid arteries of *Senp1*^SMCKO^ mice did not differ compared to that in the WT counterparts (Fig.3G).

The results above prompted us to determine a post-translational regulation of SRF. We observed increased intensity in the extra band above that for SRF protein (increased by ~15 KD) in injured carotid arteries of *Senp1*^SMCKO^ mice compared with that in WT mice (see Fig.3F). Previous report suggested that SRF could be modified by SUMO1-mediated SUMOylation^29^. To further investigate if SRF was modified by SUMOylation, carotid artery extracts obtained at 7 days after wire injury were subjected to coimmunoprecipitation assay under denaturing condition. Western blot analysis revealed a specific SUMO1conjugated SRF band that was detected in the co-immunoprecipitated proteins with anti-SRF antibody, but not with control IgG. However, SUMO2/3-conjugated SRF was not observed in the *Senp1*^SMCKO^ aortic lysates (Fig.3H). Consistently, immunostaining showed that SUMO1 expression was increased in α-SMA-positive VSMCs of carotid artery in *Senp1*^SMCKO^ mice at basal condition (Fig.S6C-D), and more dramatically increased in the injured vessels, similar to the SRF expression pattern (Fig.S6E-F). Our results indicate that *Senp*1 deficiency in VSMCs induced SUMO1-mediated SRF SUMOylation.

As vascular injury reportedly displaces SRF from the SRF-myocardin complex to form the SRF-ELK1 complex, resulting in VSMC phenotypic switch^4–7,10^, we investigated whether SRF SUMOylation affected its binding to myocardin and ELK1. To this end, VSMCs from WT and *Senp1*^SMCKO^ aortas were untreated or treated with PDGF-BB and cell lysates were subjected to co-immunoprecipitation assays under nondenaturing condition. Similar to the in vivo injury, PDGF-BB increased SRF SUMOylation which was increased by the *Senp*1 deficiency. Moreover, PDGF-BB increased SRF binding to ELK1 whereas decreased SRF binding to the myocardin in VSMCs, and these PDGF-BB responses were further augmented by the *Senp*1 deficiency. These results suggest a role of SRF SUMOylation in the SRF-ELK complex formation (Fig.3I).

We also assessed if ELK phosphorylation is required for SRF-ELK complex formation. To this end, we examined effects of AZD6244, a specific inhibitor of ELK1 activation, on the binding of SRF to myocardin and ELK1 in cultured aortic VSMCs of WT and *Senp*1^SMCKO^ mice. Indeed, AZD6244 treatment specifically blocked ELK1 phosphorylation without affecting expressions of ELK, SRF or myocardin. Co-immunoprecipitation analyses showed that AZD6244 did not affect SRF SUMOylation. However, AZD6244 drastically disrupted PDGF-BB-induced SRF-ELK1 complex while enhanced the SRF-Myocardin complex in WT mice and partially restored the association of SRF with myocardin in *Senp*1^SMCKO^ mice (Fig.3J). These data indicate that both SRF SUMOylation and ELK1 phosphorylation are required for a switch from the SRF-myocardin complex to the SRF-ELK1 complex in response to PDGF-BB stimulation.

### SRF SUMOylation regulates SRF-ELK1 complex formation

**Fig. 4.**
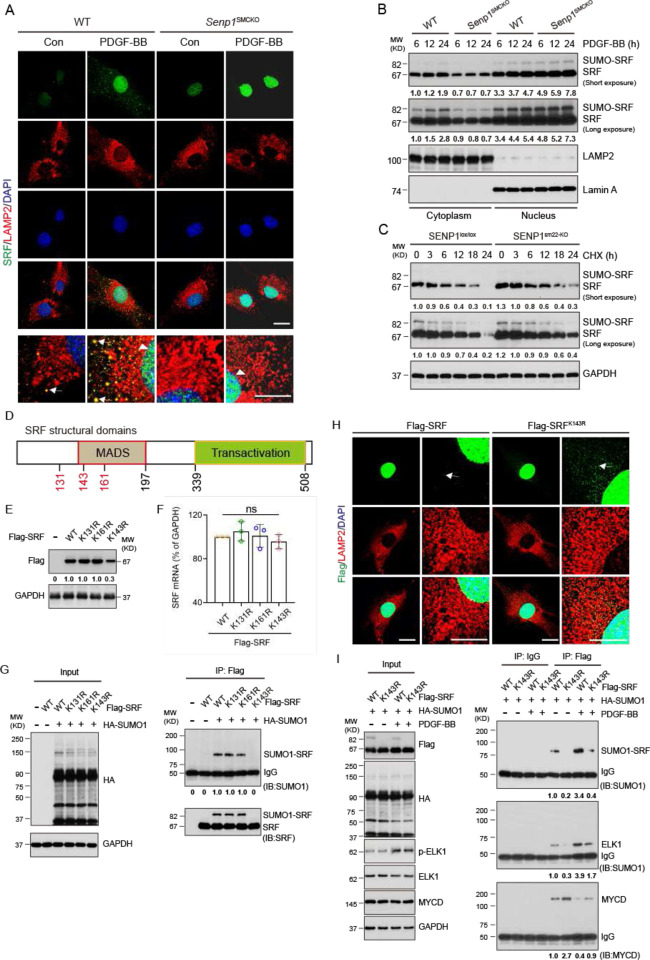
SRF SUMOylation regulates the SRF-ELK1 complex formation. **A–C**. VSMCs were isolated from un-injured WT and *Senp*1^SMCKO^ mice. (**A)**. Cells were treated with PDGF-BB (10 ng/ml) for 24 h and subjected to immunofluorescence co-staining with SRF and LAMP2. High magnification of merged images is shown at the bottom of each panel. Lysosomal and nuclear SRF are indicated by arrows and arrowheads, respectively. **(B)**. Cells were treated with PDGF-BB (10 ng/ml) for 0–24. Cytoplasm and nuclear fractions were isolated by cellular fractionation followed by western blot for SRF, lysosomal marker LAMP2 and nuclear marker lamin A. Relative protein levels are presented by taking untreated WT as 1.0 (n=3 per group). (**C)**. VSMCs were treated with CHX (100 ng/ml) for 0–24 h followed by western blot for SRF and GAPDH. Relative protein levels are presented by taking untreated WT as 1.0 (n=3). **D**. A diagram for the structural domains of mouse SRF protein, containing the DNA-binding MADS domain and the transactivation domain. The putative SUMOylation K residues are indicated. **E–I**. MOVAS-1 cells were transiently transfected with Flag-tagged SRF-WT, SRF^K131R^, SRF^K161R^ or SRF^K143R^ without (E, F, H) or with (G, I) HA-SUMO1. (**E**) Cell lysates were subjected to western blot with the anti-Flag antibody. GAPDH as the loading control (n = 3 per group). (**F**) Total RNAs were used for qRT-PCR for Srf mRNA levels. (**G)** Cells lysates were subjected to coimmunoprecipitation assays under denaturing condition with anti-Flag followed by western blot with anti-HA (SUMO1). The original input lysates were also analyzed with GAPDH as a loading control. Relative protein levels are presented by taking untreated WT as 1.0 (n=3). (**H)** Flag-SRF and Flag-SRF^K143R^ -expressing cells were subjected to immunofluorescence co-staining of Flag (green) and anti-LAMP2 (red) with DAPI counterstaining for nucleus (blue). (**I)** Flag-SRF and Flag-SRF^K143R^-expressing cells were untreated or treated with PDGF-BB for 24. Cells lysates were subjected to co-immunoprecipitation assays under non-denaturing condition with anti-Flag or IgG control followed by western blot with anti-HA (SUMO1), anti-ELK1 or antimyocardin antibodies. The original input lysates were also analyzed with GAPDH as a loading control. Relative protein levels are presented by taking untreated WT as 1.0 (n=3). Data are mean ± SEM. ** P < 0.01; ns, no significance. One-way ANOVA with Bonferroni post hoc analysis. Scale bars, 20 μm (A, I).

To determine how SUMOylation regulates SRF activity and SRF-ELK complex, we first examined if intracellular localization of SRF was altered by SENP1 deficiency in cultured aortic VSMCs. Immunofluorescence staining showed low SRF levels in normal VSMC nuclei. PDGF-BB stimulation significantly increased SRF expression in both lysosomes and nuclei, where SRF was co-localized with lysosome marker LAMP2 and nuclear marker DAPI, respectively. However, SENP1 deficiency induced SRF accumulation from the lysosomes and nuclei (Fig.4A). PDGF-BB stimulation also increased nuclear expression of KLF4, another transcription factor regulating VSMC function^28,30,31^. However, KLF4 expression and localization were not affected by SENP1 deletion (Fig.S7). We confirmed the SRF localization in SENP1deficient VSMCs by a cellular fractionation assay; PDGF-BB stimulation significantly increased total SRF and SUMOylated SRF levels in nuclear and cytoplasm factions, while SENP1 deficiency induced a profound shift of total SRF and SUMOylated SRF from the lysosomes to the nucleus (Fig.4B). Cycloheximide assays indicated that SENP1 deficiency significantly sustained the levels of both SUMOylated and total SRF proteins in cultured aortic VSMCs (Fig.4C). These results indicate that SUMOylation promoted the location change of SRF from lysosomes to nuclei, protecting SRF from degradation.

Although SUMO1-mediated SUMOylation has been previously reported for SRF, its role in the SRF regulation remains unclear^29^. Bioinformatics analyses indicated that SRF protein contains three putative SUMOylation sites (K131, K143, and K161) in the MADS domain (Fig.4D and Fig.S8). Therefore, we examined effects of KR mutations (K131R, K143R, and K161R) on SRF protein stability. In MOVAS-1 cells transfected with these mutation-carrying plasmids, similar protein levels were detected in SRF^K131R^, SRF^K161R^ mutants and wild-type SRF, whereas that of the SUMOylation deficient SRF^K143R^ mutant was approximately one-third that of wild-type SRF (Fig.4E). Notably, mRNA levels of the wild-type SRF and all three mutants did not differ (Fig.4F). We further determined SRF SUMOylation by co-immunoprecipitation assays under denaturing condition. Results revealed that the SUMO1-conjugated SRF accumulated in MOVAS-1 cells with co-expression of HA-SUMO1 and Flag-SRF, SRF^K131R^, or SRF^K161R^. However, SUMO1-conjugated SRF level was diminished in SRF^K143R^ mutant (Fig.4G). Immunofluorescence staining indicated that SRF mutation at K143 (K143R) enhanced the accumulation of SRF in the lysosomes in PDGF-BB-stimulated VSMCs (Fig.4H), supporting that SRF SUMOylation promoted a SRF translocation from lysosome to nucleus.

We then determined the effect of SRF SUMOylation on SRF-ELK1 complex formation. Coimmunoprecipitation assays revealed that the non-SUMOylated mutant SRF^K143R^ significantly increased SRF binding to myocardin concomitant with reduced binding to ELK1 in the untreated and PDGF-BB-treated VSMCs (Fig.4I). These results suggest that SRF SUMOylation at K143 induces its nuclear accumulation and switches its binding preference from myocardin to ELK1 in VSMCs in response to PDGF-BB stimulation.

### SRF SUMOylation and SRF-ELK complex regulate VSMC proliferation, migration, and phenotypic switch

**Fig. 5.**
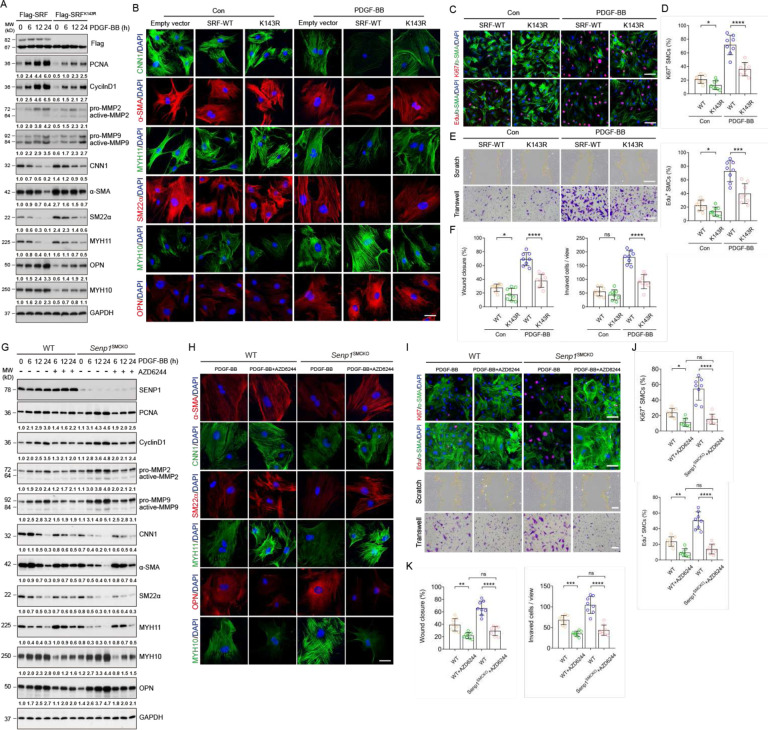
SRF SUMOylation and the SRF-ELK complex regulate VSMC proliferation, migration and phenotypic switching. **A–F.** MOVAS-1 cells were stably transfected with empty vector, Flag-SRF or Flag-SRF^K143R^ and pooled clones were expanded for the designed experiments. (**A)**. Cells were untreated or treated with PDGF-BB (10 ng/ml) for 0–24 h followed by western blot for VSMC proliferation, migration and phenotypic markers. Relative protein levels are presented by taking untreated SRF-WT as 1.0 (n=3). (**B)**. Cells were untreated or treated with PDGF-BB (10 ng/ml) for 24 h and were subjected to immunofluorescence co-staining with VSMC contractile/synthetic markers as indicated (n=3). (**C–F)**. Cells were serum-starved for 24 hours followed by treatment with PDGF-BB (10 ng/ml) for 24 h. (C) Cells were used for Edu incorporation assay or Ki67 staining with α-SMA. Representative images are presented. (D) % Ki67^+^ and Edu^+^ VMSC were quantified (n=8 per group). (E) Cells were subjected to scratch and transwell assays in the absence or presence of PDGF-BB for 24 h before fixation and imaging. Representative images are presented. (F) % wound closure and # of invaded VMSCs were quantified (n = 8 per group). **G–K**. Primary aortic VSMCs from WT and *Senp1*^SMCKO^ mice were treated with PDGF-BB (10 ng/ml) for 0–24 h in the absence or presence of AZD6244 (1 μmol/L). (**G**) Cell lysates were subjected to western blot for VSMC proliferation, migration and phenotypic markers. Relative protein levels are presented by taking untreated WT as 1.0 (n=3). (**H)**. Cells were subjected to immunofluorescence co-staining with VSMC contractile/synthetic markers as indicated (n=3). (**I–K)**. Cells were serum-starved for 24 hours and subjected to Edu incorporation, scratch and transwell assays. Representative images are presented in (I). % Ki67^+^ or Edu^+^ VMSC (J), % wound closure and # of invaded VMSCs (K) were quantified (n = 8 per group). Data are mean ± SEM. * P < 0.05; ** P < 0.01; *** P < 0.001; **** P < 0.0001; ns, no significance, using one-way ANOVA followed by Tukey’s multiple comparisons test. Scale bars, 10 μm (B, H); 50 μm (C, E, I).

To determine the functional significance of SRF SUMOylation in VSMCs, we generated stable MOVAS-1 lines overexpressing a Flag-tagged empty vector, SRF-WT, or SRF^K143R^. Western blotting confirmed that stable transfection of SRF^K143R^ enhanced the contractile marker expression (CNN1, α-SMA, SM22α and MYH11) and inhibited synthetic marker expression (MYH10 and OPN) in MOVAS-1 cells with or without PDGF-BB stimulation (Fig.5A). These observations were confirmed by immunohistochemical staining (Fig.5B). Moreover, SRF^K143R^ decreased protein levels of PCNA, CyclinD1 MMP2, and MMP9 (Fig.5A). Consistently, SRF^K143R^ inhibited proliferation and migration of MOVAS-1 cells with or without PDGF-BB stimulation, as shown by the decreased number of Edu^+^Ki67^+^ cells (Fig.5C–D), and reduced the percentage of migrating cells in both scratch and Transwell assays (Fig.5E–F). These data suggest that SRF SUMOylation at K143 plays an important function in controlling proliferation, migration, and the synthetic phenotypic switch of VSMCs.

Because ELK1 phosphorylation is required for the SRF-ELK complex formation, we next determined its role in regulating VSMC functions. Similar to the observations that in K143R-expressing cells, AZD6244 treatment in primary aortic VSMCs from WT and *Senp1*^SMCKO^ mice attenuated PDGF-BB-induced expression of cell cycle-related proteins (CyclinD1 and PCNA) and migratory proteins (MMP2 and MMP9) (Fig.5G). Moreover, western blot (Fig.5G) and immunohistochemical staining (Fig.5H) revealed that AZD6244 enhanced and suppressed contractile and synthetic marker expression, respectively. AZD6244 significantly inhibited the proliferation of PDGF-BB-stimulated aortic VSMCs from WT and *Senp1*^SMCKO^ mice, as determined by Ki67 staining and Edu incorporation assays (Fig.5I–J). Next, scratch and Transwell assays revealed that AZD6244 treatment decreased PDGF-BB-induced in vitro migration of VSMCs from WT and *Senp1*^SMCKO^ mice (Fig.5I, K). These findings suggested that inhibition of the SRF-ELK1 axis by AZD6244 attenuated PDGF-BB-induced VSMC proliferation, migration, and synthetic phenotype switch.

#### The SRF SUMOylation and phosphor-ELK were upregulated in human intimal hyperplasia.

**Fig. 6.**
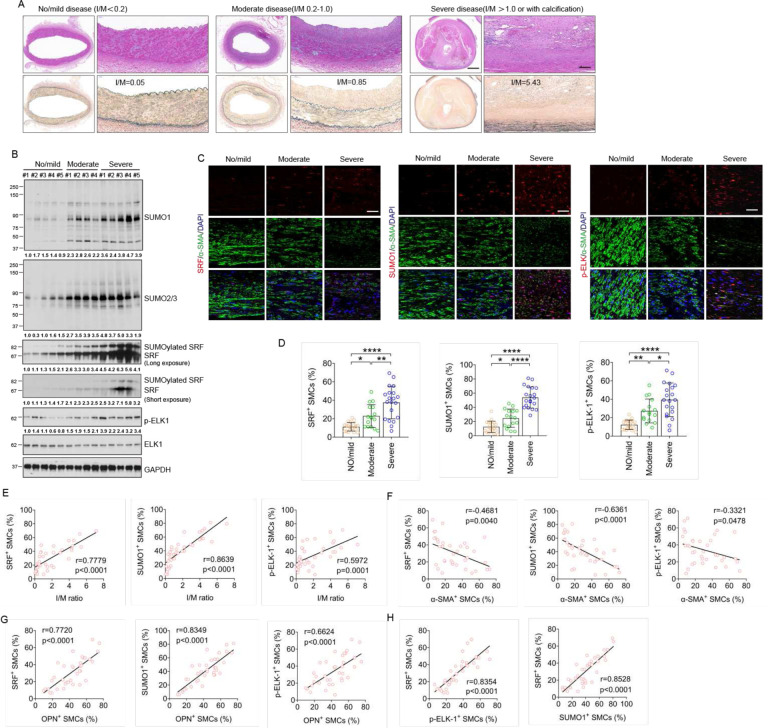
The SRF SUMOylation and phosphor-ELK were upregulated in human intimal hyperplasia. Left main coronary arteries of patients with no/mild (n = 5), moderate (n = 4), and severe CAD (n = 5) were collected for this study. **A.** Morphological assessment and classification disease severity in human left main coronary arteries. HE (upper) and EVG staining (lower). Scale bar: 1000μm (left) and 100μm (right). **B.** The protein levels of SUMO1, SUMO2/3, SRF, p-ELK1 and t-ELK1 were determined by western blot. GAPDH served as the control. Relative protein levels are presented by taking the first no/mild as 1.0. **C**. Immunocytochemical analysis of SRF, SUMO1 and p-ELK1. Five fields per section from each sample are analyzed. Representative images of immunofluorescence staining for α-SMA (green) and SRF (red), SUMO1 (red), or p-ELK1 (red). Nuclei were stained with DAPI (blue). Scale bar: 20 μm. **D.** Quantitative analysis of SUMO1, SRF or p-ELK1- positive cells to α-SMA- positive VSMCs in the lumen. Data are mean ± SEM. * P < 0.05; ** P < 0.01; **** P < 0.0001. One-way ANOVA with Bonferroni post hoc analysis. **E.** Scatter plots of SRF-, SUMO1- or p-ELK1- positive cells and the I/M ratio. The corresponding Spearman’s correlation coefficient (r) between SRF-, SUMO1- or p-ELK1- positive cells and the I/M ratio, and the P value are shown. **F.** Scatter plots of SRF-, SUMO1- or p-ELK1-positive cells and α-SMA-positive cells. The corresponding Spearman’s correlation coefficient (r) between SRF-, SUMO1- or p-ELK1-positive cells and α-SMA- positive cells, and the P value are shown. **G.** Scatter plots of SRF-, SUMO1- or p-ELK1-positive cells and OPNpositive cells. The corresponding Spearman’s correlation coefficient (r) between SRF-, SUMO1- or p-ELK1- positive cells and OPN- positive cells, and the P value are shown. **H.** Scatter plots of SRF-, SUMO1- or pELK1-positive cells. The corresponding Spearman’s correlation coefficient (r) between SRF-, SUMO1- or pELK1-positive cells, and the P value are shown. Data are mean ± SEM. Correlation analyses between variables were performed using the Pearson rank correlation test. P values were two-tailed and values <0.05 was considered statistically significant.

To determine whether the role of SRF SUMOylation in VSMC phenotypic switch observed in mouse vascular injury models translates to human CVD, we examined SUMO1, SRF, p-ELK, OPN, and α-SMA in the left main coronary arteries of patients with no/mild (grade I plaque), moderate (grade II plaque), or severe coronary artery disease (CAD) (grade III and IV plaque) (Fig.6A). Compared with no/mild CAD group, western blot results showed that protein levels of SUMO1, SRF, and p-ELK1 were markedly increased in moderate CAD group, and further increased in severe CAD group. SUMO2/3 protein levels were markedly increased in all CAD groups, but with no difference between moderate and severe groups. However, compared with no/mild CAD group, there was no increase of total ELK1 protein in moderate and severe CAD group (Fig.6B). In the same samples, immunohistochemical staining also showed that the expression of SRF, SUMO1, and p-ELK1 progressively increased with disease severity, and most of them were co-localized with α-SMApositive VSMCs (Fig.6C–D). Moreover, a strong positive linear relationship between the intima/media (I/M) ratio and number of VSMCs expressing SRF (r=0.7779, P<0.0001), SUMO1 (r=0.8639, P<0.0001) or SRF (r=0.5972, P=0.0001) was observed (Fig.6E). We next examined the contractile marker α-SMA and synthetic marker OPN expression in this patient cohort. Immunostaining revealed that luminal VSMCs exhibited increasing OPN expression and decreasing α-SMA expression with increase in CAD severity (Fig.S9A, B). α-SMA expression inversely correlated with SRF (r=−0.4681, P=0.0040), SUMO1 (r=−0.6361, P<0.0001), pELK1 (r=−0.3321, P=0.0478) (Fig.6F), OPN (r=−0.4764, P=0.0033) expression, and I/M ratio (r=−0.6219, P<0.0001) (Fig.S9C). OPN expression positively correlated with SRF (r=0.7720, P<0.0001), SUMO1 (r=0.8349, P<0.0001), p-ELK1 (r=0.6624, P<0.0001) expression (Fig.6G), and I/M ratio (r=0.6644, P<0.0001) (Fig.S9C). Finally, SRF expression positively correlated with SUMO1 (r=0.8528, P<0.0001) and p-ELK1 (r=0.8354, P<0.0001) expression (Fig.6H). These observations establish an association between the SRF SUMOylation, ELK1 phosphorylation, and CAD severity, as well as VSMC phenotypic switch in human CAD samples.

### Blocking shift from SRF-myocardin to SRF-ELK complex by AZD6244 inhibits injury-induced neointimal formation

**Fig. 7.**
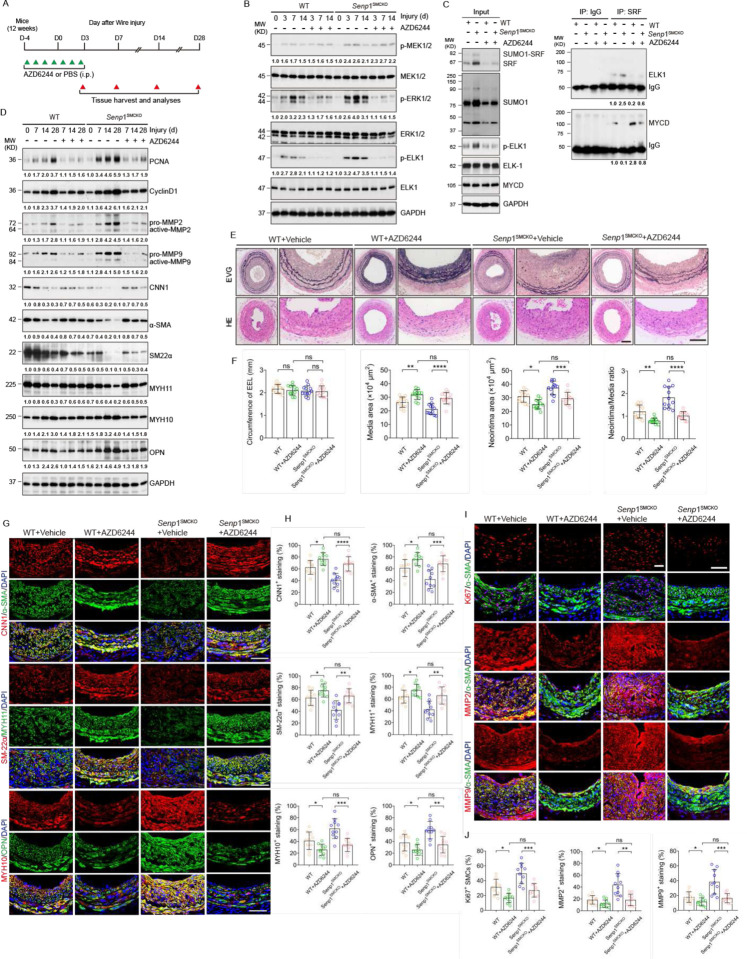
Blocking shift from SRF-myocardin to SRF-ELK complex by AZD6244 inhibits injury-induced neointimal formation. **A.** A diagram for AZD6244 injection protocol. 12-week-old WT and *Senp1*^SMCKO^ mice were received injection of AZD6244 (25 mg/kg) or vehicle (PBS) once daily from 4 days before carotid artery injury to 3 days after injury (D-4 to D3). Artery tissues were harvested on D3, D7, D14 and D28. **B.** Carotid artery tissues were subjected to western blot for the MEK-ERK-ELK1 signaling. Relative protein levels are presented by taking untreated WT as 1.0 (n=3). **C.** Carotid artery tissues were subjected to co-immunoprecipitation assays with anti-SRF followed by western blot with anti-ELK1 and anti-myocardin. Relative protein levels are presented by taking untreated WT as 1.0 (n=3). **D.** Carotid artery tissues were subjected to western blot for the VSMC proliferation, migration and phenotypic markers. Relative protein levels are presented by taking untreated WT as 1.0 (n=3). **E**. Representative photomicrographs of EVG staining and HE staining of carotid arteries harvested on day 28 post-injury from WT and *Senp1*^SMCKO^ mice. **F**. Circumference of EEL, neointimal area, media area, and neointima/media ratio in carotid arteries were measured (n=12 per group). **G–H.** Carotid artery tissues were subjected to immunofluorescence staining with VSMC contractile and synthetic markers. (G) Representative images are presented. (H) MFI of each marker within the neointimal areas were quantified (n = 10 per group). **I–J.** Carotid artery tissues were subjected to immunofluorescence co-staining of α-SMA (green) with Ki67, MMP2 or MMP9 (red) with DAPI counterstaining for nuclei (blue). (I) Representative images are presented. (J) Ki67^+^α-SMA^+^ VSMCs, MFI of MMP2 and MMP9 within the neointimal areas were quantified (n = 10 per group). Data are mean ± SEM. * P < 0.05; ** P < 0.01; *** P < 0.001; **** P < 0.001; ns, no significance. One-way ANOVA with Bonferroni post hoc analysis. Scale bars, 50 μm (E, G, I). MFI: mean fluorescence intensity.

AZD6244 drastically disrupted PDGF-BB-induced SRF-ELK1 complex while partially restored the association of SRF with myocardin (see Fig.3C). These observations prompted us to test a therapeutic potential of AZD6244 in vascular remodeling. To this end, 12-week-old WT and *Senp1*^SMCKO^ mice received injection of AZD6244 (25 mg/kg) or vehicle (PBS) once daily from 4 days prior to carotid artery injury (day 0) to 3 days after injury (D-4 to D3) as indicated (Fig.7A). We first performed a series of biochemical analyses to determine effects of AZD6244 on the SRF-ELK signaling in these mice. Although AZD6244 had no inhibitory effects on p-MEK1/2 or total ERK1/2, as previously reported^32^, it markedly reduced phosphorylation of MEK downstream targets ERK1/2 and ELK1 on days 3–14 post-surgery (Fig.7B). Importantly, phosphorylation levels of ERK1/2 and ELK1 did not differ between WT and *Senp1*^SMCKO^ mice after AZD6244 treatment, suggesting that AZD6244 abolished the effect of VSMC-specific *Senp*1 deletion mediated MEK1/2-ERK1/2ELK1 pathway in injury-induced carotid artery (Fig.7B). Of note, AZD6244 reduced SRF, SUMO1 and SUMOylated SRF at 7 days after wire injury without any effects on expressions of total ELK or myocardin (Fig.7C). However, AZD6244 dramatically attenuated the injury-induced SRF-ELK1 complex while restored SRF-myocardin complex formation in *Senp*1^SMCKO^ mice (Fig.7C).

To test whether AZD6244-mediated suppression of the SRF-ELK1 axis in VSMCs results in a corresponding reduction in SRF-ELK1 activity, we detected the transcription levels of their downstream target genes by western blot and qRT-PCR. Consistent with altered phosphorylated ELK1 levels and its binding with SRF, AZD6244 significantly reduced the expression of SRF-ELK1-mediated synthetic markers (MYH10 and OPN) but increased that of SRF/myocardin-mediated contractile markers (CNN1, α-SMA, SM22α, and MYH11) (Fig.7D). Moreover, AZD6244 treatment significantly suppressed the expression of proliferation markers (PCNA and CyclinD1), and increased the activities of migratory proteins (MMP2 and MMP9), as evident by their cleavage in injured arteries (Fig.7D). AZD6244 treatment significantly inhibited injury-induced expression of Col15a1, Col5a2, Col6a2, Timp1, Mmp14 and Col1a1 at 28 days after wire injury as determined by qRT-PCR (Fig.S10).

We then performed a series of morphological and immunohistochemical analyses to determine the effects of AZD6244 on vascular structure, VSMC proliferation, and phenotypic switch by evaluating the carotid arteries of WT and *Senp1*^SMCKO^ mice on day 28 post-injury. *Senp*1 deficiency enhanced neointima formation, and AZD6244 treatment significantly decreased the neointima areas and neointima/media ratios. However, aortic parameters between the WT and *Senp1*^SMCKO^ mice did not differ after AZD6244 treatment (Fig.7E–F), suggesting that AZD6244 abolished the effects of *Senp*1 deficiency on the vascular remodeling.

Effect of ADZ6244 on VSMC contractile and synthetic markers were then verified by immunostaining. AZD6244 treatment in *Senp1*^SMCKO^ mice suppressed the augmented expression of SRF-ELK1 downstream targets MYH10 and OPN, but rescued that of the SRF-myocardin targets CNN1, α-SMA, SM22α, and MYH11 (Fig.7G–H). Moreover, AZD6244 treatment in injured arteries significantly suppressed PCNA and cyclin D1 expression from day 7 to day 28 post-injury, consistent with the sustained inhibition of proliferation in AZD6244-treated mice visualized by Ki67 staining. The proliferative marker levels between WT and *Senp1*^SMCKO^ mice after AZD6244 treatment did not differ (Fig.7I–J). Consistent with the decreased MMP activity detected by western blot, immunohistochemical staining showed significantly decreased MMP2 and MMP9 protein levels in both WT and *Senp1*^SMCKO^ mice after AZD6244 treatment, but no significant differences in fluorescence intensities between *Senp1*^SMCKO^ mice and WT mice (Fig.7I–J). Taken together, these data suggest that AZD6244 inhibits vascular injury-induced VSMC proliferation, migration, phenotype switch, and neointimal formation in *Senp1*^SMCKO^ mice by blocking the SRF-ELK signaling pathway.

## DISCUSSION

**Fig. 8.**
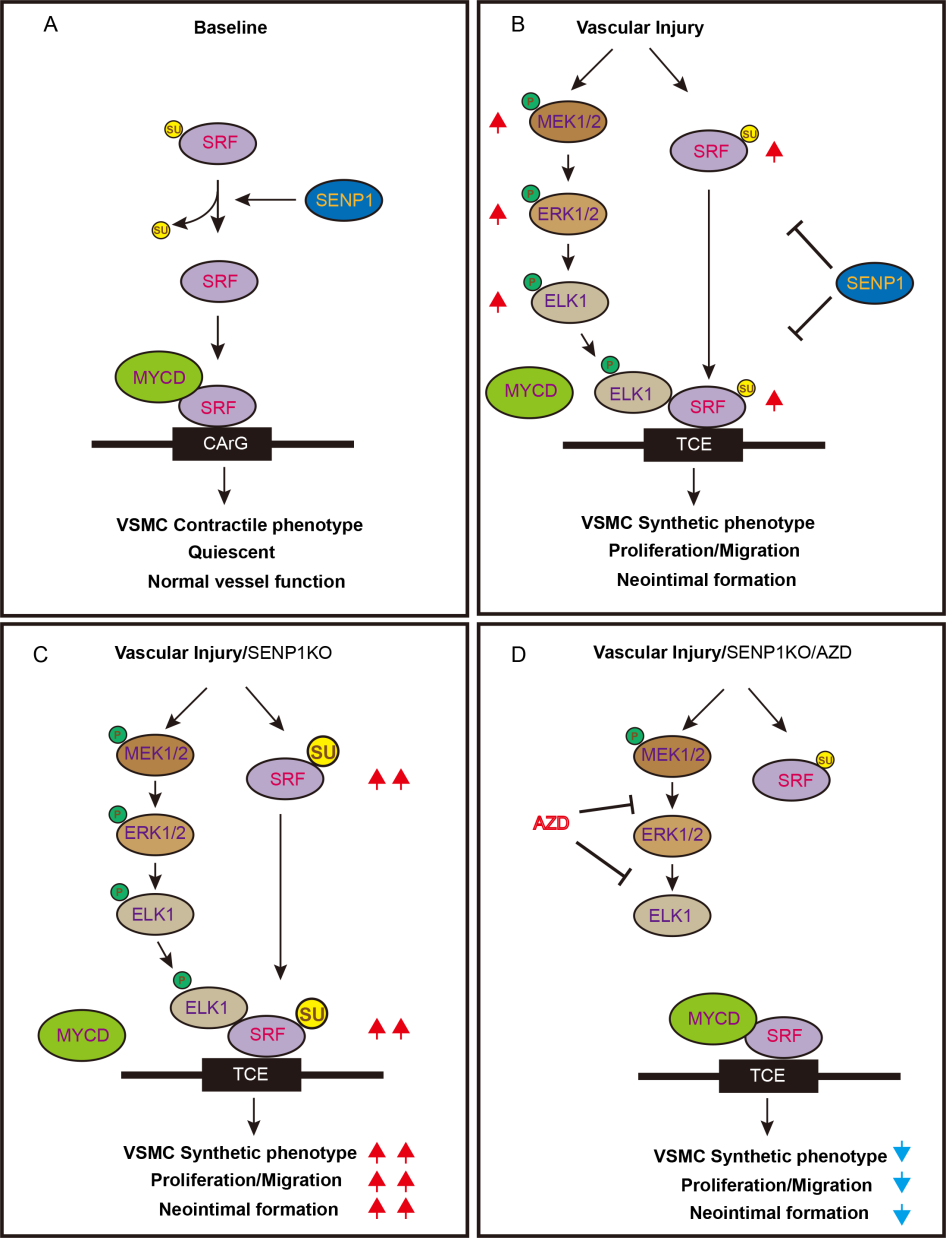
A model for the SRF SUMOylation and the SRF-ELK1 complex mediated vascular remodeling. **A**. Under normal condition, SRF is present in a deSUMOylation state and retains its binding with MYCD to maintain the VSMC contractile phenotype. **B**. In response to vascular injury, activated ELK1 and increased SUMOylated SRF form a complex and induce VSMC synthetic phenotypic switch, VSMC proliferation and migration, leading to neointimal formation. **C**. SENP1 deficiency in VSMCs dramatically increases SUMO1mediated SRF SUMOylation at lysine 143 and the SRF-ELK1 complex, which augments VSMC phenotypic switch and neointimal formation. **D**. Pharmacological inhibition of phosphor-ELK1 by AZD6244 prevents the SRF-ELK complex and/or restores the SRF-MYCD complex, attenuating the excessive proliferation, migration, and neointimal formation. AZD: AZD6244; CArG: CA(A/T)_6_G box; MYCD: myocardin; TCE: ternary complex element; Su: SUMO1.

In our present study, we report that SRF SUMOylation modulates the VSMC responses to PDGF-BB in cultured cells and to vascular injury in murine models. We demonstrate that SENP1 deficiency in VSMCs accelerates injury-induced VSMC proliferation, migration, and phenotypic switch, promoting neointimal formation and vascular remodeling. Our analyses of human CAD specimens reveal a striking correlation among SRF expression, SUMO1 level, ELK activation, VSMCs phenotypic switch, and CAD severity. Mechanistically, SENP1 deficiency in VSMCs induces SUMO1-conjugated SUMOylation of SRF at K143 and promotes its translocation from the lysosomes to nucleus, thereby increasing SRF stability in the nucleus; moreover, SUMO1-modified SRF switches its binding partner from myocardin to ELK1, accelerating VSMC proliferation, migration, and synthetic phenotype switch during vascular remodeling. These findings identify a novel function of SENP1-mediated deSUMOylation in orchestrating the complex process of vascular remodeling. Importantly, pharmacological inhibition of ELK1 activity by AZD6244 prevents the shift from SRFmyocardin to SRF-ELK complex, attenuating VSMC proliferation, migration, and neointimal formation (Fig.8), thus providing a potential therapeutic target for CVD treatment.

Notably, SUMOylation of other protein targets in VSMCs have been reported. SUMOylated KLF4 reportedly plays an important role in PDGF-BB-induced VSMC proliferation by reversing the transactivation of KLF4 on p21^33^. In our study, KLF4 protein expression and nuclear localization were not altered by SENP1 deficiency. It is unclear if SENP1 regulates KLF4 complex formation in VSMC. Alternatively, KLF4 SUMOylation is regulated by other SENPs. SUMOylation of PKD2 channels regulates its membrane recycling and is necessary for intravascular pressure-induced arterial contractility^34^. Similarly, SUMOylation of Runx2 (Runtrelated transcription factor) in VSMCs causes Runx2 degradation, thereby preventing atherosclerotic calcification in mouse models^35^. Further studies are needed to examine whether SENP1-mediated deSUMOylation regulates PKD2 or Runx2 in VSMCs during vascular remodeling. A key finding of the present study is that SUMO1-mediated SRF SUMOylation is increased in PDGF-BB-treated VSMCs and in wire injury-induced mouse carotid arteries, and is further augmented by SENP1 deficiency. Moreover, increased SUMO1 levels and SRF SUMOylation were correlated with VSMC phenotypic switch from contractile to synthetic state. Under SENP1 deficiency, SUMOylation induces high stability of SRF protein and alters its binding partners in VSMCs. Our study suggests that the SRF SUMOylation at K143 controls the SRF-ELK complex formation and VSMC phenotypic switch. SRF, the key mediator of gene transcription and function in VSMCs^6,10^, functions with two families of signal-regulated cofactors. Of these, three ternary complex factors (TCFs)-ELK1, Net, and SAP1-are regulated by Ras-ERK signaling, while the myocardin/MRTFs are regulated by the Rho-actin pathway^4^. Our study reveals that SRF SUMOylation plays a critical role in mediating the SRF complex competition and VSMC phenotypic switch. We provide the following evidence: 1) SENP1 deficiency in VSMCs induced SUMO1-mediated SRF SUMOylation, increased SRF protein level, but not mRNA level, in mouse aorta and isolated VSMCs; SRF SUMOylation promoted SRF localization from the lysosomes to the nucleus, protecting SRF against degradation. 2) SENP1 deficiency in VSMCs promoted SRF binding switch from SRF-myocardin to SRF-ELK1 complex, resulting in VSMC conversion from contractile to synthetic state; and 3) Moreover, SUMOylation at K143 of SRF, together with ELK1 phosphorylation, are required for SRF-ELK1 complex formation and subsequent VSMC phenotypic switch. Importantly, preventing the shift from SRF-myocardin to SRF-ELK complex by AZD6244 attenuates neointimal formation in *Senp*1-deficient mice.

The neointimal formation, a hallmark of vascular remodeling in CVD, is associated with a significant increase in VSMC migration, proliferation, and phenotypic switch^36^. The clinical significance of our study is that we established abnormal protein SUMOylation and phosphor-ELK in human intimal hyperplasia, and demonstrated its correlation with VSMC phenotypic switch and neointimal formation. A few studies have shown that post-translational modification contributes to the initiation and progression of intimal hyperplasia. Notably, protein SUMOylations have been proposed to play a key role in the promoting of VSMC proliferation and may provide potential targets for treatment and prevention of intimal hyperplasia^25,37–39^. However, to date, there are no reports showing a direct association between SUMOylation and VSMC fate in human intimal hyperplasia. Our findings in human CAD specimens demonstrated that the SRF SUMOylation and the SRFELK1 complex serve as a link between PDGF-BB/vascular injury and neointimal formation. We showed that SRF, SUMO1, and phosphor-ELK1 levels increased in VSMCs on PDGF-BB stimulation, and SENP1 deficiency augmented these responses. Moreover, SRF, SUMO1, and phosphor-ELK1 levels were higher in luminal VSMCs from CAD groups compared with the normal coronary arteries, progressively increasing with the disease severity. Accordingly, VSMCs in human intimal hyperplasia exhibited phenotypic switches (with gain of synthetic markers and loss of contractile markers) and correlated with the expression of SRF, SUMO1, and phosphor-ELK1 and the I/M ratios.

An important clinical implication of our study is that we provided a proof-of-concept for CAD treatment with AZD6244 by inhibiting the SRF-ELK1 axis while restoring the SRF-myocardin complex. A pharmacological MEK1/2 inhibitor, U0126, reportedly blocked the phosphorylation of ELK1, prevented PDGFBB-induced association of ELK1 and SRF, and promoted SRF binding to myocardin, thus attenuating the suppressive effects of PDGF-BB on expression of contractile phenotype-related genes^40^. Local or systemic administration of another MEK1/2 inhibitor, PD98059, was found to suppress ERK1/2 activation, its target gene expression, and neointima formation in carotid artery injury model^41,42^. These reports indicate that MEK1/2 inhibition may represent a therapeutic strategy for the prevention of neointima formation. AZD6244 is a highly selective non-ATP-competitive MEK1/2 inhibitor that inhibits ERK1/2 phosphorylation without inhibiting p38α, MKK6, EGFR, ErbB2, or B-Raf^43,44^. We demonstrate that AZD6244 specifically blocked the ERK1/2-ELK1 pathway. Importantly, AZD6244 disrupted the SRF-ELK1 complex and promoted SRFmyocardin complex formation, corresponding to suppression of VSMC synthetic phenotype and attenuated neointimal formation. Thus, our study demonstrates that the SRF complex switch is an essential mechanism regulating VSMC phenotypic switch and neointimal formation associated with vascular injury and provides an attractive target for pharmacological treatment of CVD.

## ONLINE METHODS

### Clinical specimens

Human coronary arteries were obtained from cardiac transplant recipients with chronic rejection undergoing re-transplantation, cardiomyopathy recipients undergoing first-time transplantation, and organ donors without cardiac disease. All procedures involving human samples complied with the principles outlined in the Declaration of Helsinki and were approved by the Conjoint Health Research Ethics Board of the First People’s Hospital of Changzhou. A consent waiver was approved for surgical patients, and written informed consent was obtained from a family member of the deceased organ donors. Our procurement techniques have been described previously^45,46^. Briefly, for each arterial sample procured in the operating room, disease was macroscopically diagnosed by an experienced cardiac surgeon. Half of each sample was formalin-fixed immediately for paraffin-embedding and the other half was stored at −80 °C. The baseline characteristics of the patients are listed in Supplemental Table 1.

### Animal study

Mice were housed in the animal care facility of Yale University under standard pathogen-free conditions (temperature, 20–24 °C; relative humidity, 45–65) with a 12 h light/dark schedule and provided with food (Charles River Laboratory) and water ad libitum. Mice were cared for in accordance with the National Institutes of Health guidelines, and all procedures were approved by the Yale University Animal Care and Use Committee. Equal numbers of male and female adult mice were used in all experiments. We used a previously generated conditional gene knockout mouse model lacking *Senp1* specifically in VSMCs (*Senp1*^SMCKO^)^24^. We used EC-specific *Senp*1 deletion line (*Senp*1^ecKO^) as control^22–25^. The carotid arteries of C57BL/6, *Senp*1^lox/lox^ (WT) and *Senp*1^SMCKO^ mice (both having C57BL/6 background for more than six generations) were genotyped by western blot, quantitative RT-PCR, and immunofluorescence staining.

### Carotid artery wire injury mouse model

Wire injury was performed in *Senp*1^lox/lox^ (WT) and *Senp*1^SMCKO^ mice (10–12-weeks-old) as described previously^27^. Briefly, mice were anaesthetized by intraperitoneally injecting ketamine (80 mg/kg) and xylazine (5 mg/kg). The left carotid artery was carefully dissected, under a dissecting microscope, through a midline neck incision. The external carotid artery was ligated with an 8−0 suture immediately proximal to the bifurcation point. Vascular clamps were applied to interrupt the internal and common carotid arterial blood flow. A transverse incision was made immediately proximal to the suture around the external carotid artery. A guidewire (0.38 mm diameter; No. C-SF-15–15; Cook, Bloomington, USA) was then introduced into the arterial lumen towards the aortic arch and withdrawn ten times with an angular rotating motion. After carefully removing the guidewire, the vascular clamps were removed and blood flow was restored. Finally, the skin incision was closed. A similar procedure without wire injury on the right common carotid artery was performed to serve as control. The common carotid artery tissues were collected at specific time points after surgery for morphological and biochemical analyses.

### AZD6244 treatment

AZD6244 (Selumetinib, Catalog No. S1008, Selleckchem, TX, USA) was dissolved in 0.5% hydroxypropyl methyl cellulose and 0.4% polysorbate (Tween80). WT and *Senp1*^SMCKO^ mice were intraperitoneally injected with freshly prepared AZD6244 (25 mg/kg) daily for one week (four days before wire injury and three days after)^47^. Aortic VSMCs isolated from WT and *Senp1*^SMCKO^ mice or MOVAS-1 cells were serum-starved for 24 h and stimulated with human PDGF-BB (10 ng/mL; Catalog #220-BB; R&D Systems, MN, USA) for different periods with or without AZD6244-pretreatment (0.5 μmol/L) for 12 h^48^. Thereafter, VSMCs or MOVAS-1 cells were harvested for RNA and protein extraction or immunostaining. DMSO served as a vehicle control.

### Electrocardiogram (ECG) and blood pressure

ECG and blood pressure of the experimental animals were recorded as previously described^49^}. Briefly, animals were anesthetized by isoflurane inhalation (2.5%) in a chamber and maintained by constant 1.0% isoflurane flow through a nose cone during the procedure. A heating pad and an infrared heat lamp maintained their body temperature at 36 ± 0.5 °C. Three-lead ECG recordings were performed to determine a variety of time intervals and conduction velocities during isoflurane anesthesia. O_2_ was used to power the anesthesia system. ECG signals were collected by the Powerlab system (PL3516, AD Instruments, Dunedin, New Zealand) with LabChart software (LabChartv8.1.16), which was also used for data acquisition and analysis. For blood pressure recording, aortas were catheterized via the right carotid artery using a 1.4-F Mikro-Tip pressure catheter (Millar Instruments), and blood pressure was measured using a PowerLab system with LabChart software.

### Histological and morphometric analyses

Mice were sacrificed by an intraperitoneal injection of ketamine (80 mg/kg) and xylazine (5 mg/kg). Common carotid artery tissues were perfused and fixed with 4% paraformaldehyde for 10 min, embedded in paraffin, and 6 μm-thick sections were used for elastic van gieson (EVG) or hematoxylin and eosin (HE) staining after deparaffinization and rehydration. Serial cross-sections (6 μm) of the entire region (300 μm) at the bifurcation site of the left carotid artery were obtained. The normal carotid artery thickness was determined based on the circumference of external elastic lamina (EEL), luminal area, and media area. The extent of neointimal formation 28 days after injury was determined based on the circumference of EEL, intima, media, and intima/media ratio. Images were quantified with Image-Pro Plus software (version 6.0, Media Cybernetics, MD, USA) by a single observer who was blinded to the treatment protocols.

Left main coronary artery sections were stained with HE and EVG, and images were obtained for one section per block at a final magnification of 40×. ImageJ software (NIH, Bethesda, MD, USA) was used for morphometric analyses. Intimal (I) and media (M) thickness were measured, and their ratio and plaque stages were used to grade the severity of atherosclerosis. The means of these parameters from four different areas for each specimen were calculated. Left main coronary arteries with an I/M ratio < 0.2 were considered to have no or mild disease; 0.2–1 were considered to have moderate disease; and > 1 or with calcification were considered to have severe disease.

### Aortic ring assay

An aortic ring assay was performed as described in our previous studies^45,46^. The dissected aortas were cut into cylindrical, 3-mm-long segments. The rings were suspended by two tungsten wires mounted in a vessel myograph system (Danish Myotechnologies, Aarhus, Denmark). The aortas were bathed in oxygenated Krebs buffer and subjected to a resting tension of 9.8 mN. After 60-min equilibration with frequent washings, aortic rings were contracted with phenylephrine (PE) at 10^−9^–10^−4^ M and 3×10^−7^ M. PE (10^−9^–10^−4^ M) + Lnitroarginine methyl ester (L-NAME, 300 μM) or L-NAME (300 μM), and their relaxation response to acetylcholine (Ach; 10^−9^–10^−4^ M) or sodium nitroprusside (SNP, 5×10^−10^–5×10^−7^ M).

### VSMCs isolation and culture

Primary aortic VSMCs were isolated from WT and *Senp1*^SMCKO^ mice according to elastase/collagenase digestion protocols^50^ with slight modifications. Briefly, thoracic aortas were carefully isolated and dissected away from connective tissues under a light microscope. The isolated aortas were digested in HBSS containing 1 mg/mL collagenase A (10103578001, Sigma-Aldrich, MO, USA) for 5 min at 37 °C to promote adventitia removal. The denuded vessels were incubated with 0.5 mL Dulbecco’s Modified Eagle’s medium (DMEM, Thermo Fisher, MA, USA) containing 10% fetal bovine serum (FBS, Life Technologies, NY, USA), 1.5 mg/mL collagenase A, 0.5 mg/mL elastase (LS006365, Worthington, NJ, USA), and antibiotics at 37 °C for 30 min, while the digest was triturated with a pipette every 15 min. The mixture was centrifuged, then the cells were resuspended in DMEM supplemented with 10% FBS and 1% penicillin-streptomycin (PenStrep, GIBCO-Invitrogen, NY, USA), and cultured in 35-mm dishes in a CO_2_ incubator at 37 °C. After the primary cells reached 90% confluency, they were digested, and the lysate was used for western blot analysis. Earlypassage (passage 3–4) VSMCs were used in all experiments.

MOVAS-1 cell line (Cat: #ATCC^®^CRL-2797^™^), derived from aortic smooth muscle cells of male C57BL/6J mice, was purchased from American type culture collection (ATCC; Manassas, VA, USA). MOVAS-1 cells were cultured in DMEM (Sigma-Aldrich, St. Louis, MO, USA) supplemented with 10% FBS and 1% penicillinstreptomycin, and maintained at 37 °C in a 5% CO_2_ incubator.

### Bulk RNA-sequencing and gene expression analysis

Total RNA extracted from aorta samples of WT and Senp1^SMCKO^ mice at basal or at 14 days after wire injury was subjected to bulk RNA-seq analysis. RNA integrity was assessed using the RNA Nano 6000 Assay Kit on the Bioanalyzer 2100 system (Agilent). Sequencing libraries were generated using the rRNA-depleted RNA by NEBNext^®^ Ultra^™^ Directional RNA Library Prep Kit from Illumina^®^ (NEB, USA), following the manufacturer’s instructions. The libraries were sequenced on an Illumina Hiseq 4000 platform, and bp pairedend reads were generated. The raw reads were assessed for quality using FastQC and mapped to the reference genome using HISAT2 (v2.0.4), and gene expression levels were quantified using Cuffdiff (v2.1.1). Differential expression analyses were performed using the DESeq2 R package. Genes with an absolute fold change (FC) >1.5 and a false discovery rate (FDR) <0.05 were considered as differentially expressed genes. Volcano plot was generated using the open sources R language. Heat maps were generated by MEV (MultiExperiment Viewer) program. Gene Ontology (GO) analysis was carried out with the DAVID 6.8 online analysis system (https://david.ncifcrf.gov/home.jsp).

### Examination of proliferative ability

We assessed VSMC proliferation using 5-ethynyl-2′-deoxyuridine (Edu) incorporation assay and Ki67 staining. For Edu incorporation assay, cells were incubated with 10 μM Edu for 4 h before fixing in 4% paraformaldehyde for 30 min at 37 °C. The fixed cells were assayed using the Click-iT Edu kit (C10646, ThermoFisher Scientific, MA, USA). For Ki67 staining, Cells were fixed, permeabilized, and subjected to Ki67 staining according to the manufacturer’s instructions. Images were captured by LSM880 laser confocal microscope (Carl Zeiss, Germany). Data are presented as the ratio of Edu-or Ki67-positive cells to total αSMA-positive VSMCs.

### Examination of migratory ability

In vitro migratory activity of VSMCs was measured using a scratch assay. VSMCs (1×10^5^ cells/well) were seeded in six-well plates and cultured in DMEM supplemented with 10% FBS and 1% penicillin-streptomycin. At 90% confluence, the growth medium was replaced with DMEM supplemented with 0.2% FBS. After 24-h starvation, a wound was created by scraping the cell monolayer with a 200 μL sterile pipette tip across the center of the well. We further assessed VSMC migratory activity using Transwell migration assay. VSMCs (1.5×10^4^ cells/well) were plated in the upper Transwell chamber. The pore size of insert was 8 μm. After 24h culture, the VSMCs that migrated to the lower chamber were fixed with methanol and stained with 1% crystal violet. These migrated VSMCs were quantified in four randomly selected areas at 100× magnification using Cellsens Dimention 1.15 software (Olympus, Tokyo, Japan).

### Plasmids and transfection

HA-SUMO1 was purchased in Addgene (Cambridge, MA, USA). To obtain Flag-SRF, full-length SRF (mouse, Locus ID 20807) was purchased from ORIGENE (CAT#: TL502142) and cloned into p3xFlag-Myc-CMV-24 vector. Various Flag-SRF mutations (K143R, K131R, K161R) were generated using SUMOylation prediction websites (SUMOplot, JASSA, and GPS-SUMO) and primers were listed in Supplemental Table 2. MOVAS-1 cells (~80% confluent) were transfected with the desired plasmids using Lipofectamine 2000 following the manufacturer’s instructions. In most experiments, equimolar ratios of plasmids were used for co-transfection experiments, and the total amount of plasmids was adjusted with the empty vector. To ensure that the various plasmids express comparable levels of the proteins, a two-fold amount of Flag-SRF^K143R^ plasmid, as compared to wild-type Flag-SRF, was used for transfection in some experiments.

### Real Time-PCR

Total RNA from mouse carotid artery tissue or cells was isolated using TRIzol reagent (Thermo Fisher). Purified RNA (500 ng) was reverse transcribed using PrimerScript RT Reagent Kit (Takara, Japan) and 1 μg of cDNA was used to perform quantitative RT-PCR with LightCycler 480 Real-time PCR system (Roche) in accordance with the manufacturer’s instructions. Primers are listed in Supplemental Table 2.

### Immunofluorescence staining

Cells (1×10^5^ cells/well) were seeded in glass-bottomed culture dishes, fixed with freshly prepared 4% paraformaldehyde for 15 min, and permeabilized with 0.2% Triton X-100 in PBS for 5 min. The paraffinembedded mouse carotid artery sections (6 μm-thick) and paraffin-embedded human coronary artery sections were deparaffinized and rehydrated. After permeabilization with 0.5% Triton X-100 in PBS for 5 min, the sections or cells were incubated with primary antibodies overnight at 4 °C. Normal isotype IgG was used as negative control. After washing with PBS, the samples were incubated with secondary antibodies for 2 h at 37 °C in the dark. Nuclei were labeled with DAPI, and sections were visualized using an LSM880 laser confocal microscope (Carl Zeiss, Germany). Antibodies used in this study are listed in Supplemental Table 3.

### Protein extraction and western blot analysis

Human or murine arterial tissue or in vitro cell lysates were prepared using 1× cell lysis buffer (Cell Signaling Technologies, MA, USA) containing protease inhibitors (Cat. 04693159001, Sigma-Aldrich, MO, USA). As the protein concentration from a single mouse carotid artery tissue was low, we mixed tissue samples from three to six individuals for protein extraction. The extracted proteins were electrophoresed on 10% SDSpolyacrylamide gel electrophoresis and transferred to a polyvinylidene difluoride membrane (0.45 μm, BioRad, CA, USA). Each membrane was blocked with 5% nonfat dry milk in Tris-buffered saline and 0.1% Tween 20 (TBST) for 2 h and then incubated with the primary antibody at 4 °C overnight. The membrane was washed and incubated with the horseradish peroxidase-conjugated secondary antibody in 5% non-fat milk in TBST buffer for 2 h. Antibodies are listed in Supplemental Table 3. Proteins were detected using a Chemiluminescence Detection Kit (sc-2048, Santa Cruz, TX, USA) and band intensities were quantified by densitometry with ImageJ software. Uncut gels were provided in Fig.S11.

### Co-immunoprecipitation (Co-IP) assay

The tissues or cells were lysed in lysis buffer A (30 mM Hepes pH 7.6, 100 mM NaCl, 0.5% Nonidet P-40, and protease inhibitor mixture) on ice for 10 min, centrifuged at 4 °C for 10 min at 13,000 rpm, and 500 μg of cell lysate was incubated with 5 μg of the indicated primary antibodies at 4 °C overnight. Anti-IgG served as the negative control. The immune complexes were purified with 20 μL protein A/G agarose at 4 °C for 6 h, centrifuged, washed with ice-cold cell lysis buffer, and analyzed by immunoblotting. The antibodies used are listed in Supplemental Table 3.

### Cycloheximide (CHX) assay

To assess protein stability, cells were seeded into 12-well plates at a density of 2×10^5^ cells/well, cultured for 24 h, and transfected with indicated plasmids. After treatment with CHX (100 ng/mL) for indicated time periods, cells were harvested and steady-state target protein levels were determined by western blot.

### Nuclear and cytoplasmic preparations

Cells were fractionated using NE-PER Nuclear and Cytoplasmic Extraction Reagents (Cat. 78833; Thermo Fisher) according to manufacturer’s instructions to obtain nuclear and cytoplasmic fractions. Briefly, cells were washed with PBS, incubated with cytoplasmic extract reagent I for 10 min, followed by ice-cold cytoplasmic extract reagent II, and centrifuged for 5 min to obtain the cytoplasmic extract. The insoluble fraction was incubated with ice-cold nuclear extract reagent for 40 min and centrifuged for 10 min to obtain the nuclear extract.

### Statistical analyses

Group sizes were determined by a priori power analysis for a two-tailed, two-sample Student’s t-test with an α of 0.05 and power of 0.8, in order to detect a 10% difference in lesion size at the endpoint. All quantifications (artery morphometrical analyses, immunofluorescence intensity, protein and mRNA levels) were performed in a blind fashion. All figures are representative of at least three experiments unless otherwise noted. The data were analyzed using the GraphPad Prism, version 8.0.1 (GraphPad Software, Inc.) and SPSS, version 20.0 (SPSS Inc.). All graphs report mean ± standard error of mean (SEM) values of biological replicates. The normality and variance were not tested. Comparisons between two and more than two groups were performed by unpaired two tailed Student’s t-test and one-way ANOVA followed by Tukey’s multiple comparisons test or by two-way ANOVA followed by Sidak’s multiple comparisons test, respectively, using Prism 8.0.1 software (GraphPad). Correlation analyses between variables were performed using the Pearson rank correlation test. Two-tailed P values < 0.05 were considered statistically significant.

## ABBREVIATIONS:

CHX: cycloheximide
Co-IP: co-immunoprecipitation
CVD: cardiovascular disease
DAPI: 4′,6-diamidino-2-phenylindole
EBD: Evans blue dye
EC: endothelial cell
ECG: electrocardiogram
Edu: 5ethynyl-2′-deoxyuridine
EEL: external elastic lamina
ELK1: Ets-like transcription factor-1
EVG: elastic van gieson
H&E: hematoxylin and eosin
KD: knockdown
KO: knockout
MRTF: myocardin-related transcriptional factor
MYCD: myocardin
PBS: phosphate-buffered saline
PDGF-BB: platelet-derived growth factor-BB
PFA: paraformaldehyde
Senp1: SUMO specific peptidase-1
siRNA: small interfering RNA
SMA: smooth muscle a-actin
SRF: serum response factor
SUMO: small ubiquitin-like modifier
TCE: ternary complex element
VSMCs: vascular smooth muscle cells
